# A case report of acute liver failure caused by biliary ascariasis in children

**DOI:** 10.3389/fped.2026.1809021

**Published:** 2026-04-15

**Authors:** Jiaqi Li, Liting Hou, Qianqian Liu, Bin Yan, Weikai Wang, Hongxia Gao

**Affiliations:** 1 Department of the First Clinical Medical College, Gansu University of Chinese Medicine, Lanzhou, Gansu, China; 2 The Second Department of the PICU, Gansu Maternal and Child-Care Hospital, Lanzhou, Gansu, China; 3 The Second Department of the Neonatology, Gansu Maternal and Child-Care Hospital, Lanzhou, Gansu, China

**Keywords:** acute liver failure, ascariasis, biliary tract diseases, children, jaundice

## Abstract

Most patients with biliary ascariasis present with acute abdominal pain and biliary colic. Typically, conservative treatment involving deworming agents is employed. However, for those who do not respond to pharmacological therapy, endoscopic or surgical intervention becomes necessary. Generally, the mortality rate is low, and the prognosis is favorable. This case describes a pediatric patient exhibiting symptoms of intestinal disease, including upper abdominal pain and jaundice, resulting from extrahepatic biliary tract obstruction caused by roundworms. This condition subsequently led to complications such as obstructive jaundice and acute liver failure. Abnormal signals within the gallbladder cavity were detected through imaging techniques, including abdominal magnetic resonance cholangiopancreatography (MRCP) and enhanced abdominal computed tomography (CT). These abnormalities typically manifest as strip-shaped, tortuous, and tubular filling defects, thereby confirming the diagnosis of biliary ascariasis. Following the oral administration of albendazole for deworming and DPMAS treatment for acute liver failure, the child's condition improved, and he was discharged from the hospital. Subsequent follow-up indicated no recurrence of the disease. The objective of this study is to facilitate the early identification of biliary ascariasis in children, actively manage associated complications, and prevent the development of severe conditions.

## 1 Introduction

Biliary ascariasis is a parasitic disease resulting from roundworms infiltrating the biliary system. This condition remains relatively prevalent in developing countries and regions with inadequate sanitation. Roundworms possess the ability to bore through tissues. When the host experiences stimuli such as fever, infection, malnutrition, or improper deworming, the worms may retrograde through the duodenal papillae, penetrating the biliary tract or pancreas, which can lead to severe complications including biliary obstruction, cholangitis, and pancreatitis (1). Due to their anatomical characteristics, particularly the relatively narrow bile ducts, and their underdeveloped immune systems, children often exhibit more severe clinical manifestations of biliary ascariasis. Most children present with sudden, paroxysmal, draping-like pain in the right upper abdomen, accompanied by nausea and vomiting. Some may also develop obstructive jaundice, fever, and other signs of cholangitis (2). Most cases of biliary ascariasis can be effectively managed with antispasmodic and analgesic treatment, deworming therapy, and endoscopic deworming, resulting in a favorable prognosis (3). However, instances of acute liver failure attributable to biliary ascariasis are exceedingly rare. The potential mechanisms underlying acute liver failure in this context include complete obstructive jaundice caused by the worms obstructing the biliary tract, cholestatic liver injury, secondary bacterial cholangitis, and systemic inflammatory response syndrome, among others. The interplay of these factors can precipitate extensive necrosis of liver cells within a short timeframe (4, 5). Notably, in non-endemic regions or medical institutions with limited clinical experience, the tendency for worms to “escape” detection during ultrasound and magnetic resonance cholangiopancreatography examinations due to peristalsis results in a relatively high rate of missed diagnoses of biliary ascariasis in imaging (6). This article presents clinical data from a case of acute liver failure resulting from biliary ascariasis in a child. Through a review of the literature, it examines the clinical characteristics, diagnosis, and treatment of this condition, as well as the lessons learned. The aim is to improve clinicians’ understanding of the complications associated with biliary ascariasis and to offer guidance for early identification and timely intervention.

### 1.1 Clinical data

The patient, a 13-year-old female, was admitted to the hospital primarily due to “upper abdominal distension and pain for one week and yellowing of the skin and sclera for five days.” One week prior to admission, the child experienced upper abdominal distension and pain of unclear origin, which intensified after meals and was accompanied by nausea and dizziness. Five days before admission, she had a single episode of fever, with a peak temperature of 38.6℃. Following self-treatment with various oral medications (specific drugs and dosages unknown), her upper abdominal distension and pain showed minimal improvement, and she developed jaundice, dark yellow urine, and grayish-white stool. One week after the child's upper abdominal distension and pain, she visited the local health center, where blood tests revealed: WBC: 3.9 × 10^9/L, N%: 70%, PLT: 79 × 10^9/L; blood biochemistry indicated ALT at 1,766 U/L and AST at 998 U/L. An abdominal ultrasound showed slightly thickened liver parenchyma with enhanced echoes and edema of the gallbladder wall. Subsequently, the patient was referred to our hospital for further evaluation of “liver dysfunction under investigation: 1. Viral hepatitis? 2. Drug/toxic liver damage?” and was admitted for treatment. Physical examination reveals the patient is conscious, exhibiting jaundice in the skin, mucous membranes, and sclera. The abdomen is soft with mild tenderness noted in the upper region, but there is no rebound tenderness. The liver and spleen are palpable approximately 0.5 cm below the ribs, and there is notable percussion pain in the liver area. A positive Murphy's sign is observed.

## 2 Laboratory data

**Figure 1:**
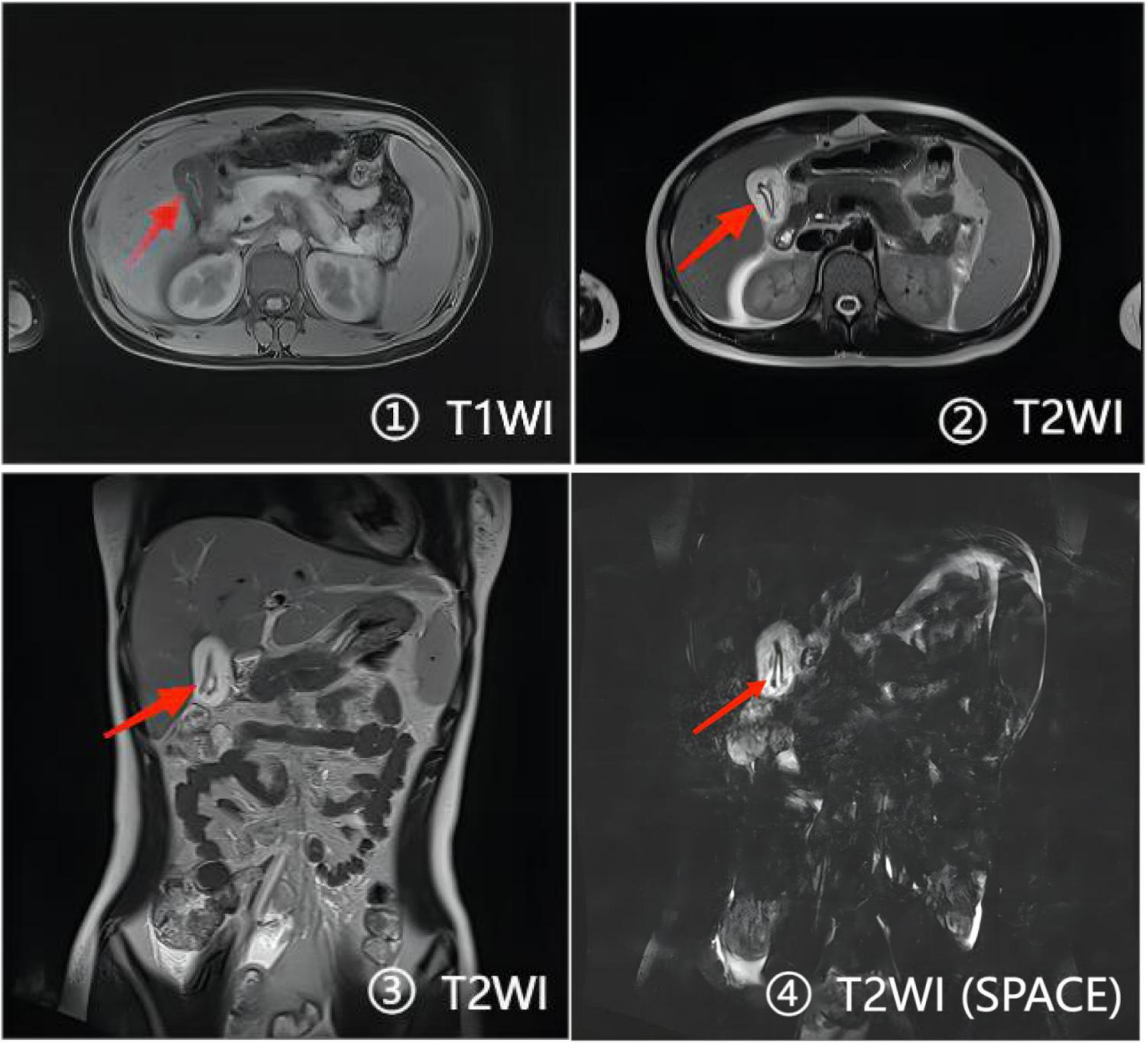
The gallbladder wall thickens, and abnormal signals like curves are seen in the lumen. T1W1 shows a low signal in the middle and a high signal at the edge, and T2W1 shows a high signal in the middle and a low signal at the edge.

Blood routine test on the first day of hospitalization, revealed the following: white blood cells at 4.35 × 10^9/L, red blood cells at 4.64 × 10^12/L, neutrophils at 65.3%, platelets at 74 × 10^9/L, and eosinophils at 0.2%. Coagulation function tests indicated an activated partial thromboplastin time (APTT) of 42.6 s, prothrombin time (PT) of 25.1 s, fibrinogen (FIB) at 0.9 g/L, international normalized ratio (INR) of 2.18, and D-dimer at 1.08 mg/L. The determination of antithrombin III activity was 34.2. Blood ammonia levels were measured at 76.1 µmol/L. Blood biochemistry results included alanine aminotransferase at 1,449.1 U/L, aspartate aminotransferase at 900.5 U/L, albumin at 32.9 g/L, and total bile acid at 215.1 µmol/L. Total bilirubin was recorded at 14.8 mg/dL, with direct bilirubin at 10.3 mg/dL and indirect bilirubin at 4.5 mg/dL. Alpha-fetoprotein levels were noted at 28.04 ng/mL. Testing for hepatitis A, B, C, and E antibodies returned negative results. No abnormalities were found in serum amylase, serum lipase and urine amylase. Upper abdominal magnetic resonance imaging (MRI) and magnetic resonance cholangiopancreatography (MRCP) (see Figure 1) showed abnormal signals in the gallbladder cavity, with roundworms frequently considered as a potential cause. Inflammatory manifestations of the gallbladder, abnormal signals in the liver, and a small amount of effusion in the Glisson sheath surrounding the liver were also observed. Abdominal color Doppler ultrasound indicated edema and thickening of the gallbladder wall.

## 3 Treatment and outcome

The child patient was admitted for pertinent tests and examinations, which indicated that the patient had obstructive jaundice, cholangitis and acute liver failure. Treatment included polyene phosphatidylcholine and adenosylmethionine butandisulfonate to protect the liver and enhance bile secretion, along with nutritional support. On the second day of admission, a complete upper abdominal magnetic resonance imaging (MRI) and magnetic resonance cholangiopancreatography (MRCP) showed abnormal signals in the gallbladder cavity, raising the suspicion of roundworm infestation. Inflammatory manifestations of the gallbladder were noted, prompting inquiries into the family's medical history. The child had ingested oral insecticides 20 days prior to admission. On the third day of hospitalization, albendazole tablets (0.2 g per tablet, 2 tablets each time, once a day) were orally administered for 1 days. A follow-up enhanced abdominal CT scan indicated that the gallbladder wall was edematous and thickened, yet no roundworms were detected. During the treatment period, the patient received two intravenous infusions of human albumin and four component blood transfusions, including frozen plasma and cryoprecipitate, to address abnormal coagulation function and a progressive decrease in albumin levels. DPMAS (Double Plasma Molecular Adsorption System) treatment was administered twice to manage concurrent acute liver failure. After 13 days of hospitalization, subsequent re-examinations of blood biochemistry, blood ammonia, complete blood count, and coagulation function revealed normalization of all abnormal indicators. An abdominal CT scan indicated the absence of roundworms. The patient reported no abdominal pain, and significant improvements were noted in the skin, mucous membranes, and sclera compared to prior assessments. After 15 days of hospitalization, the patient was discharged following recovery. Two weeks following the patient's discharge from the hospital, routine fecal tests conducted at the outpatient department revealed no presence of parasite eggs. Additionally, routine blood tests, along with assessments of liver and kidney function (including transaminase and bilirubin levels), showed no abnormal indicators. Similarly, the routine fecal tests of the patient's family members also yielded no evidence of parasite eggs.

## 4 Discussion

Due to inadequate personal hygiene practices, roundworms complete their development in the intestinal tract following oral ingestion, with adult worms residing in various regions of the gastrointestinal tract. Research indicates (7) that the infection rate of roundworms in children aged 2 to 3 years increases, peaking between the ages of 8 and 14, while the infection rate in individuals aged 15 and older gradually declines. This disease is often identified when patients excrete worms during defecation or through fecal analysis. Ascaris typically inhabit the jejunum; however, they exhibit a propensity to migrate and seek small openings throughout the human intestinal tract. When they enter the bile duct via the Oddi sphincter or ascend along the bile duct to colonize the gallbladder or liver parenchyma (8), the body may exhibit non-specific symptoms, including right upper abdominal pain, vomiting, and jaundice. In some cases, this can lead to severe complications (9), such as intestinal obstruction, cholecystitis, cholangitis, liver abscess, and acute liver failure.

The primary manifestations of all types of jaundice include yellowing of the skin, mucous membranes, and sclera. However, distinct types of jaundice exhibit characteristic signs and laboratory findings. In this case, the patient presented with obstructive jaundice (10), which was marked by dark yellow urine and terracoidal stool. Blood biochemistry revealed a significant elevation in direct bilirubin levels. An abdominal magnetic resonance imaging (MRI) combined with magnetic resonance cholangiopancreatography (MRCP) examination indicated the presence of roundworms in the gallbladder. The resulting cholestasis obstructed the flow of bile into the duodenum, a condition referred to as cholestatic jaundice, highlighting an issue with the bilirubin excretion pathway. Nevertheless, the imaging findings suggested that the roundworms had not completely occluded the gallbladder cavity, leading to the hypothesis that the obstructive jaundice in this patient was incomplete. However, if the obstruction becomes severe, it may result in increased pressure within the gallbladder, retrograde infection, and significant damage to liver cells. Obstructive jaundice must be distinguished from two other types of jaundice: 1. Pre-hepatic jaundice (hemolytic jaundice), which arises from the excessive destruction of red blood cells and the resultant overproduction of bilirubin, primarily characterized by elevated levels of unconjugated bilirubin that surpass the liver's metabolic capacity. Associated conditions include hereditary spherocytosis, extensive hematoma absorption, and autoimmune hemolytic anemia. 2. Hepatocellular jaundice, which occurs when liver cells are damaged, leading to a diminished capacity to process bilirubin. This type is frequently observed in cases of viral hepatitis, alcoholic liver disease, and drug-induced liver injury.

During the hospitalization, the patient's temperature was measured three times daily. The patient had experienced fever symptoms only prior to admission and exhibited no fever symptoms during the hospital stay. Following the identification of the cause and type of jaundice, the patient's medical history was further scrutinized, particularly regarding the administration of oral deworming medications taken 20 days prior to admission.（Following the identification of the cause and type of jaundice, the patient's medical history was further scrutinized, particularly regarding the administration of oral deworming medications taken 20 days prior to admission） It is plausible that the prior dosage of these medications was inadequate or that a significant number of intestinal roundworms were present, leading to their “irritation” and subsequent entry into the biliary tract. Some literature indicates (11) that deworming agents are generally contraindicated during the active migration of roundworm larvae in the lungs, as this may elevate the risk of severe pneumonia. Additionally, if a substantial number of parasites are present in the intestines, deworming medications may precipitate intestinal obstruction. Roundworms exhibit a propensity for “burrowing,” and whether deworming drugs can exacerbate this behavior, facilitating their entry into the biliary tract, necessitates further investigation. Typically, patients experiencing acute biliary obstruction are advised against the use of anthelmintic medications due to the potential exacerbation of the condition. Nevertheless, in this instance, the imaging findings revealed partial rather than complete occlusion of the gallbladder lumen by the roundworm. Following an assessment of the patient's physiological parameters, anthelmintic therapy was initiated. Two days following deworming, the patient's abdominal magnetic resonance imaging (MRI) re-examination revealed the absence of roundworms. However, despite symptomatic treatment with medications such as cholagmites, liver-protective agents, and enzyme-lowering drugs, the child's condition deteriorated rapidly. Upon admission for further tests and examinations, abnormal indicators were identified, including elevated blood ammonia levels, impaired coagulation function, hypoalbuminemia, and hepatosplenomegaly, indicating the onset of acute liver failure. Furthermore, during the course of treatment, bilirubin levels continued to rise progressively, prompting the initiation of DPMAS therapy. DPMAS denotes a dual plasma molecular adsorption system (12). Plasma, isolated by a plasma separator or a centrifugal blood cell separator, sequentially flows through an anion exchange resin bilirubin adsorber and a neutral macroporous adsorption resin hemopfusion device. During this process, toxins, including bilirubin, bile acids, and inflammatory factors, are adsorbed. The treated plasma, now combined with formed elements such as blood cells, is subsequently returned to the body. In this case, although the roundworms did not completely occupy the gallbladder cavity, they were capable of inducing bile stasis and the formation of bile sludge. Partial biliary obstruction resulted in a decreased bile flow rate, which promoted the development of bile sludge and further intensified the obstruction (13). The onset of acute liver failure arises from a confluence of factors rather than solely from mechanical obstruction of the biliary tract. Complications, including acute cholangitis, systemic inflammatory response, and ischemic hepatitis, contribute to this condition. Notably, bile acid-induced hepatocyte toxicity occurs when long-term cholestasis leads to the accumulation of hydrophobic bile acids, such as chenodeoxycholic acid, within liver cells. This accumulation can directly induce liver cell death through oxidative stress and apoptotic signaling pathways (14, 15). The severity of this process is closely linked to the duration of obstruction, while its correlation with the degree of obstruction is minimal. Imaging examinations provide a snapshot of the anatomical state at a specific moment, whereas the patient's pathophysiological condition is subject to continuous change. As the disease progresses, an initially incomplete obstruction may evolve into a functionally complete obstruction due to the movement of the worm's position. Consequently, when a patient experiences liver failure, the true extent of obstruction may significantly surpass what is detectable through imaging studies. Following two DPMAS treatments, the child's transaminase, bilirubin, and other indicators exhibited significant reductions. Following the onset of acute liver failure, it is essential to identify the underlying cause for effective treatment, in addition to providing symptomatic care based on abnormal indicators. For instance, antiviral therapy with nucleoside (acid) drugs is necessary in cases of hepatitis virus infection. In instances of drug-induced liver injury, all suspected medications must be discontinued. For patients diagnosed or suspected of mushroom poisoning, the administration of penicillin G and silymarin should be considered. If the therapeutic response to the underlying cause is inadequate, options such as artificial liver support or liver transplantation should be explored. This case indicates that the severity of biliary ascariasis should not be underestimated solely based on the “incomplete obstruction” identified through imaging. In patients who have developed obstructive jaundice, cholangitis, or liver failure, it is crucial to consider endoscopic intervention or alternative approaches, even when complete biliary obstruction is not evident on imaging. Such measures are necessary to alleviate potential functional obstruction and facilitate bile drainage, thereby preventing further deterioration of the patient's condition (16).

Biliary ascariasis is typically diagnosed using ultrasound (17) or MRCP examination. However, as the eggs produced by the worms traverse the biliary tract and intestines, numerous cases may be overlooked. This condition can be effectively treated with oral deworming medications. In cases where patients do not respond to oral treatment, removal of the worms may be necessary through endoscopy (18) or surgical intervention. In this case, the deworming effect was observed one days after the re-administration of albendazole (11). No roundworms were detected upon re-examination through imaging; however, the child developed complications, including acute liver failure and obstructive jaundice. This underscores the necessity for clinicians in non-endemic areas or regions with atypical clinical manifestations (19) to remain vigilant for similar symptoms of this disease. Once a diagnosis is established or highly suspected, it is essential to conduct close follow-up abdominal ultrasound examination during deworming treatment to screen for potential complications, such as biliary obstruction, secondary infections, and liver function impairment. Timely intervention measures should be implemented to prevent the onset of critical conditions.

## Data Availability

The original contributions presented in the study are included in the article/supplementary material, further inquiries can be directed to the corresponding author.
